# Sheep’s Clothing

**Published:** 1871-05

**Authors:** 


### SHEEP'S CLOTHING.

THAT is, appearing to be a harmless, innocent sheep, when in reality the character is wolfish, destructive, sanguinary. Parents should be on their guard as to the character of the newspapers, magazines and books which come into their families, just as it is their duty to be on their guard to prevent unwholesome, decayed, or poisonous provisions from being placed on their tables.

However valuable and well conducted any publication may be in the main, if now and then articles are admitted which ridicule the Bible, its religion, its ministers, and its Sabbath, or call their truth in question, even indirectly, such publication should be banished from the house. Poison is not the less fatal because it is enveloped in sugar-candy ; the more able a publication is, the greater mischief it is capable of doing, if it uses the influence it has gained over its readers in a way to undermine their faith in the Christian system. When that is done as to a daughter, it is but the taking hold of her hand to lead her into the street ; and the average life of the street-walker is less than five years ! When you undermine your son's faith in religion, he takes his first step toward the street, toward late hours, evil associations, the theatre, the club-house, the billiard-room, and the card-table ; then follow intemperance, disease, a squandered fortune, and an early death.

The influence of the daily papers of the largest cities, as a class, are antagonistic of Bible religion ; the writers for them, being, to too large an extent, young men of education from England and Ireland, who have thrown off parental restraints, and, in the ambition to rise in the new world, sacrifice on that altar, virtue, temperance, honor, religion, and everything else. A large number of the writers for the New York press, of superior ability, die early, by violence, drunkenness, or debauchery, led to these things by night work and the potations connected with it ; by Sabbath work and its compellings from religious worship; by introductions into the greenrooms of theatres, and the known facilities of debauchery presented there. Just in proportion as these young men have superior education and talents and genius, can these be used against good morals and religion, under the perverting influences of wine and women, of gaming and gluttony.

A most fascinating writer, in a weekly pictorial which enters fifty thousand families fifty-two times a year, especially commending itself to our daughters by its magnificent plates of fashions and patterns, teaches that beer and all spirituous liquors are fattening; if, then, a young lady wants to be a little plumper, rather more of a butter-ball and rather less of a scraggle or bag of bones, all that she has to do is to sip whiskey and guzzle lager.

More recently another weekly pictorial, in an article written on a health subject, by a talented physician, ridicules an article of faith held by all Christendom. The lesson of the article is, —

TAKE HEED WHAT YE READ.

Let Christian parents at least have an eye to the character of the newspapers and magazines which come into their families.

